# Low selection of HIV PrEP refills at private pharmacies among clients who initiated PrEP at public clinics: findings from a mixed-methods study in Kenya

**DOI:** 10.1186/s12913-024-10995-0

**Published:** 2024-05-11

**Authors:** Katrina F. Ortblad, Alexandra P. Kuo, Peter Mogere, Stephanie D. Roche, Catherine Kiptinness, Njeri Wairimu, Stephen Gakuo, Jared M. Baeten, Kenneth Ngure

**Affiliations:** 1 Public Health Sciences Division, Fred Hutchinson Cancer Center, 1100 Fairview Ave N, Seattle, WA 98109 USA; 2 Department of Pharmacy, University of Washington, Seattle, WA USA; 3 Center for Clinical Research, Kenya Medical Research Institute, Nairobi, Kenya; 4 Department of Global Health, University of Washington, Seattle, WA USA; 5 Department of Medicine, University of Washington, Seattle, USA; 6 School of Public Health, Jomo Kenyatta University of Agriculture and Technology, Nairobi, Kenya

**Keywords:** Client preferences, Pre-exposure prophylaxis (PrEP), HIV prevention, Differentiated service delivery (DSD), Private pharmacies, Kenya

## Abstract

**Background:**

In Africa, the delivery of HIV pre-exposure prophylaxis (PrEP) at public healthcare clinics is challenged by understaffing, overcrowding, and HIV-associated stigma, often resulting in low PrEP uptake and continuation among clients. Giving clients the option to refill PrEP at nearby private pharmacies, which are often more convenient and have shorter wait times, may address these challenges and improve PrEP continuation.

**Methods:**

This mixed methods study used an explanatory sequential design. At two public clinics in Kiambu County, Kenya, clients ≥ 18 years initiating PrEP were given the option to refill PrEP at the clinic where they initiated for free or at one of three nearby private pharmacies for 300 Kenyan Shillings (~ $3 US Dollars). The providers at these pharmacies (pharmacists and pharmaceutical technologists) were trained in PrEP service delivery using a prescribing checklist and provider-assisted HIV self-testing, both with remote clinician oversight. Clients were followed up to seven months, with scheduled refill visits at one, four, and seven months. The primary outcomes were selection of pharmacy-based PrEP refills and PrEP continuation. Following pilot completion, 15 in-depth interviews (IDIs) with clients who refilled PrEP were completed. We used descriptive statistics and thematic analysis to assess study outcomes.

**Results:**

From November 2020 to November 2021, 125 PrEP clients were screened and 106 enrolled. The majority (59%, 63/106) of clients were women and the median age was 31 years (IQR 26–38 years). Over 292 client-months of follow-up, 41 clients (39%) refilled PrEP; only three (3%) at a participating pharmacy. All clients who completed IDIs refilled PrEP at clinics. The reasons why clients did not refill PrEP at pharmacies included: a preference for clinic-delivered PrEP services (i.e., pre-existing relationships, access to other services), concerns about pharmacy-delivered PrEP services (i.e., mistrust, lower quality care, costs), and lack of knowledge of this refill location.

**Conclusions:**

These findings suggest that clients who initiate PrEP at public clinics in Kenya may have already overcome barriers to clinic-delivered PrEP services and prefer PrEP access there. To reach new populations that could benefit from PrEP, a stand-alone model of pharmacy-delivered PrEP services may be needed.

**Trial registration:**

ClinicalTrials.gov: NCT04558554 [registered: June 5, 2020].

**Supplementary Information:**

The online version contains supplementary material available at 10.1186/s12913-024-10995-0.

## Background

Despite the provision of free highly effective HIV prevention interventions, including oral pre-exposure prophylaxis (PrEP) [1, 2], the rates of HIV incidence persist above the level of epidemic control in many African countries [3, 4]. Individuals that could benefit from PrEP services often face barriers to accessing PrEP at public healthcare clinics, which has resulted in low PrEP initiation and continuation (i.e., refills) in these settings [5–8]. Barriers to PrEP access include long travel distances to and wait times at the clinics, fears of stigma associated with visiting HIV care centers at the clinics, and limited hours of clinic operations [9, 10]. The delivery of PrEP services (e.g., HIV testing, counseling, drug dispensing) at private pharmacies may address some of these barriers, as pharmacies are ubiquitous in many African countries, provide diverse services, and are often open for extended hours [11–13]. Additionally, private pharmacies are often the first places many people in low- and middle-income countries seek health services [14–16], as they already provide many sexual and reproductive health-related products (e.g., treatment for sexually transmitted infections), and clients are willing to purchase products at pharmacies that are available for free at public clinics (e.g., contraception) [17].

Recent pilot studies that tested different models of PrEP initiation at private pharmacies in Kenya found that uptake was high and direct delivery of PrEP in these settings may engage populations (e.g., men, unmarried people) who could benefit from PrEP and differ from those engaged in clinic-based PrEP services [18–20]. Additionally, these studies found that PrEP continuation at private pharmacies was comparable, or in some cases higher, than that at public clinics [18, 19]. However, regulatory barriers (e.g., restrictions on cadres of workers who can prescribe antiretrovirals and complete HIV rapid diagnostic testing [RDT]) exist in Kenya and many similar settings that make the scale-up PrEP initiation at private pharmacies challenging.

Compared to PrEP initiation, PrEP refilling is less medically complex, and potentially more feasible to delivery in non-clinical settings, including private pharmacies. With PrEP refills, there are fewer concerns about dispensing PrEP during the acute HIV infection stage – as seroconversions are rare among regular PrEP users [21] – and the medical safety of PrEP has already been determined at initiation. Thus, policy makers may feel more comfortable having individuals interested in PrEP services initiate at a clinic and have the option to refill PrEP at a nearby pharmacy, so that at initiation an RDT-certified clinician or nurse with prescribing privileges is avaliable and any necessary laboratory testing (e.g., creatinine testing) can be completed. We used mixed methods to understand the feasibility of this differentiated PrEP service delivery model in Kenya and identify weak points for model refinement.

## Methods

### Study design and setting

We conducted a mixed methods study using an explanatory sequential design [22]. First, we implemented a one-arm, prospective pilot study testing a model of clinic-based PrEP initiation with the option of pharmacy-based PrEP refills (ClinicalTrials.gov: NCT04558554), then we completed in-depth interviews to better understand the pilot findings. The study was conducted in Kiambu County, Kenya, where the population-level HIV prevalence is 4% [23] and there are ~ 100 public clinics delivering PrEP (~ 10–15% of all clinics in the county) and > 6,000 registered private pharmacies [24].

For the pilot, we engaged two public clinics and three private pharmacies. The clinics selected for participation were ones our research team has previously engaged with on other PrEP implementation projects [1, 21, 25–28]; thus, they were familiar with participation in research activities. The pharmacies selected were registered with the Kenya Pharmacy and Poisons Board, had a full-time licensed pharmacist or pharmaceutical technologist, and had a private room for counseling and the provision of HIV testing services. All engaged clinicians completed a brief training on how to offer PrEP refills to new PrEP clients at nearby participating pharmacies. All engaged pharmacy providers (i.e., pharmacist and pharmaceutical technologists) completed a two-day virtual training on pharmacy-delivered PrEP services (implementation occurred during the COVID-19 pandemic) [19]. This training included counseling on HIV risk and prevention interventions, PrEP use and safety, provider-assisted HIV self-testing, drug dispensing, and record keeping; following training, on-site technical assistance was available, as needed.

### Participants and procedures

With the help of trained healthcare providers, we recruited individuals initiating PrEP at the participating clinics and enrolled those ≥ 18 years old who were not pregnant or breastfeeding and willing to engage in research activities. At the clinics, participants received PrEP services in accordance with Kenya’s national PrEP delivery guidelines [29], which include counseling on HIV risk reduction and drug adherence, HIV rapid diagnostic testing, serum creatine testing (if available), syndromic assessment of sexually transmitted infections, and a one-month PrEP supply at initiation and three-month supply at follow-up visits. At the end of each PrEP initiation or follow-up visit, participants were given the option to return to the clinic where they initiated PrEP for free refills (i.e., 0 Kenyan Shilling [KES] client fee) or alternatively refill PrEP at one of three nearby pharmacies for a 300 KES fee (~ $3 US Dollars [USD]). To facilitate pharmacy-based PrEP refilling, participants were verbally given directions to and the contact numbers of participating pharmacies; information that was written and shared with participants upon request.

Participants who opted to refill PrEP at a pharmacy were attended by trained pharmacy providers who implemented a care pathway for pharmacy-delivered PrEP services our research team developed in collaboration with Kenyan stakeholders [30] (model details reported elsewhere [19, 30]). Pharmacy providers attended to PrEP clients using a standardized prescribing checklist that guided them through conducting a behavioral HIV risk assessment, medical safety assessment, and HIV testing; clients who met the criteria on the checklist for PrEP continuation were dispensed a three-month PrEP supply. Any participants who did not meet the checklist criteria (i.e., because they reported a history of liver or kidney disease, or tested HIV-positive) were referred back to the public clinic where they initiated PrEP for further evaluation. If providers had any questions about clients’ PrEP eligibility or PrEP dispensing, remote clinicians were available for support 24/7 via phone and SMS. In this study, the HIV test kits and PrEP drugs used at pharmacies were donated by the Kenya Ministry of Health. Thus, the fee clients paid for pharmacy PrEP refills (determined after consultation with participating pharmacy providers) was to compensate providers for their time spent delivering PrEP services.

The study protocol was reviewed and approved by the Scientific Ethics Review Unit at the Kenya Medical Research Institute and the Human Subjects Division at the University of Washington. All procedures were followed in accordance with the relevant guidelines and regulations (e.g., Declaration of Helsinki). All participants provided written informed consent and received 500 KES (~ $5 USD) as compensation for their time completing research activities.

### Data collection

Research assistants stationed at the clinics and pharmacies completed questionnaires with participants following each PrEP visit (questionnaires included in Additional files 1 & 2). The questionnaires collected information on clients’ socio-demographic characteristics, healthcare seeking behaviors, sexual behaviors, self-reported PrEP adherence (follow-up visits only), and experiences and perceptions of pharmacy-delivered PrEP services. At PrEP initiation, participants were asked to report their preferred location for accessing PrEP (i.e., private clinic, public clinic, or private pharmacy). Research assistants did not participate in the delivery of PrEP services; they were only engaged in research-related activities.

### Utilization outcomes

Our primary pilot study outcomes were selection of pharmacy-based PrEP refills and PrEP continuation among enrolled participants. We defined selection of pharmacy-based refills as the percentage of participants that went to a pharmacy and were dispensed PrEP. We defined PrEP continuation as the percentage of participants due for a scheduled PrEP follow-up visit (at one, four, and seven months) who returned to a clinic or pharmacy and were dispensed PrEP [19, 21]. Additionally, we measured PrEP adherence (secondary outcome) using a validated 100-point scale that averaged participants’ self-reported responses to three questions: their number of pills missed, ability to use PrEP, and frequency of PrEP use in the past month [31]. The latter two of these two adherence questions were assessed using 5-point Likert scales; all question responses were transformed to 100-point scales, with higher scores indicating better adherence [31].

### Implementation outcomes

At each PrEP clinic or pharmacy visit, we assessed participants’ perceived acceptability of, appropriateness of, and willingness to pay for pharmacy-based PrEP refills. To assess acceptability, we asked participants how strongly they agreed (5-point Likert scale) with two statements that assessed if they liked or would recommend pharmacy-delivered PrEP services (based on the Theoretical Framework of Acceptability [32, 33]). To assess appropriateness (only measured at follow-up visits), we asked participants how strongly they agreed (5-point Likert scale) with two statements that assessed how well pharmacy-delivered PrEP services fit or were a good match for their needs (based on the Intervention Appropriateness Measure [34]). We defined outcome success for these measures as > 80% of participants agreeing or strongly agreeing with a statement. When considering willingness to pay for pharmacy-based PrEP refills, participants were asked to consider a package of services that included counseling, a medical safety assessment, HIV testing, and a three-month PrEP supply.

### Quantitative analyses

We used descriptive statistics to report all findings from the pilot study. For PrEP continuation, this outcome was only reported among participants eligible for PrEP follow-up visits; due to the short duration of the pilot study, not all enrolled participants were eligible for follow-up visits at four and seven months. To better understand PrEP continuation by participants’ preferred PrEP access location, we completed a subgroup analysis that assessed this outcome among participants who reported a preference for PrEP access at a clinic (private or public) versus a pharmacy (private) at enrollment. To determine if there were any significant differences (*p* < 0.05) in PrEP continuation between these subgroups, we used a chi-squared test. We used StataSE 16 (College Station, USA) to complete all quantitative analyses.

We aimed to enroll 200 participants in this pilot study. Based on our experience conducting other pilot studies [20, 35], this sample size was considered sufficient for generating preliminary data on our primary study outcomes. Additionally, this sample size was determined feasible given the time and budget constraints of the study.

### Factors influencing PrEP refill location

Post-pilot completion, we opted to conduct in-depth interviews with participants to better understand why hardly any selected to refill PrEP at a pharmacy (see study Results). Our sampling frame included all participants who refilled PrEP at least once during the study period, but not at the pharmacy; capturing a population we know had interest in continuing PrEP services. Given the tightly scoped nature of our research question, we anticipated that 15 interviews would be sufficient to reach thematic saturation [36]. We therefore purposefully invited eligible participants until the 15 participants were interviewed. Using semi-structured interview guides (found in Additional file 3), an experienced Kenyan qualitative researcher solicited information about interviewees’ understanding of the pharmacy PrEP refill option, what motivated their decision to refill PrEP at the clinic instead of a pharmacy, and potential barriers to refilling PrEP at a pharmacy. All interviews were conducted in a private room (at the local research team’s office or the clinic where clients were receiving PrEP services) in the interviewee’s preferred language (English or Swahili). All interviews were audio recorded, transcribed verbatim, and translated (if applicable) to English.

We analyzed all interview transcripts using thematic analysis [37]. One author (AK) achieved data immersion by reading all transcripts multiple times, then created a codebook of facilitators to clinic-based and barriers to pharmacy-based PrEP refills. AK created one spreadsheet per code, collated relevant passages from each transcript, then read through each code’s contents in its entirety. We organized codes into primary reasons for opting to refill PrEP at the clinic (as opposed to a pharmacy) and checked these for face validity with the qualitative researcher who conducted the interviews (author NW). Lastly, we selected illustrative quotes for each reason identified. To complete our qualitative analyses, we used Microsoft Excel (Redmond, USA).

## Results

From November 2020 to November 2021, we screened 125 clients initiating PrEP at public clinics and enrolled 106 participants in the pilot study. We stopped enrollment prior to achieving our target sample size because selection of pharmacy PrEP refills was much lower than anticipated (described below). Among enrolled participants, 59% (63/106) were women and the median age was 31 years (interquartile range [IQR] 26 to 38 years), Table 1. Most (67%, 71/106) participants were married and half (49%, 52/106) were in an HIV serodifferent relationship. When asked their preferred setting for PrEP refills at enrollment, clients were split among (private) pharmacies (45%, 48/106) and clinics (public: 42%, 44/106; private: 13%, 14/106).

**Table 1:** Characteristics of clients initiating PrEP at a public health clinic, *N* = 106

Characteristic	N (%)
Facility enrollment	
* Facility A*	29% (31/106)
* Facility B*	71% (75/106)
Age, median (IQR)	31 (26–38)
< *25 years*	17 (16%)
Sex	
* Female*	63 (59%)
* Male*	43 (41%)
Years of school, median (IQR)	15 (12–16)
Currently in school	18 (17%)
Married	71 (67%)
Relationship status	
*Single*	0
*Casual partner(s) only*	19 (18%)
*One primary partner only*	54 (51%)
*One primary* + *casual partners*	33 (31%)
Monthly individual income in KES, median (IQR)^a^	10000 (0–15000)
In a known serodifferent relationship	52 (49%)
Stated preferred location for accessing PrEP	
*Preferred private clinic*	14 (13%)
*Preferred public clinic*	44 (42%)
*Preferred retail pharmacy*	48 (45%)
Was seeking an SRH service^b^	104 (98%)
Uses LARC or hormonal form of contraception^c,d^	31 (29%)
Has used emergency contraception ≥ 2 times in lifetime^d^	19 (18%)
Trying to conceive	16 (15%)
Has ever tested for HIV	99 (93%)
*Months since last test, median (IQR)*	3 (1–8)
Has ever tested for HIV with primary partner	67 (63%)
Knows someone using PrEP	32 (30%)

*Abbreviations: LARC* Long-acting forms of contraception, *IQR* Interquartile range, *SRH* Sexual and reproductive health service

^a^USD equivalent is $56.40 ($0-$141). Converted from KES to USD using conversion rate averaged from 11/2020 to 12/2021 ($1USD = 110.72 KSH)—https://www.exchangerates.org.uk/KES-USD-spot-exchange-rates-history-2020.html

^b^SRH services sought included PrEP (63%, *n* = 181), HIV self-testing (20%, *n* = 57), family planning (13%, *n* = 38), pregnancy testing (3%, *n* = 10), or sexual performance enhancing drug (3%, *n* = 9)

^c^Included the following forms of contraception: implant (7%, *n* = 21), injectable (5%, *n* = 13), oral pill (3%, *n* = 8), and intrauterine device (IUD) (1%, *n* = 3)

^d^Reported among female participants only (*n* = 124)

**Fig. 1:**
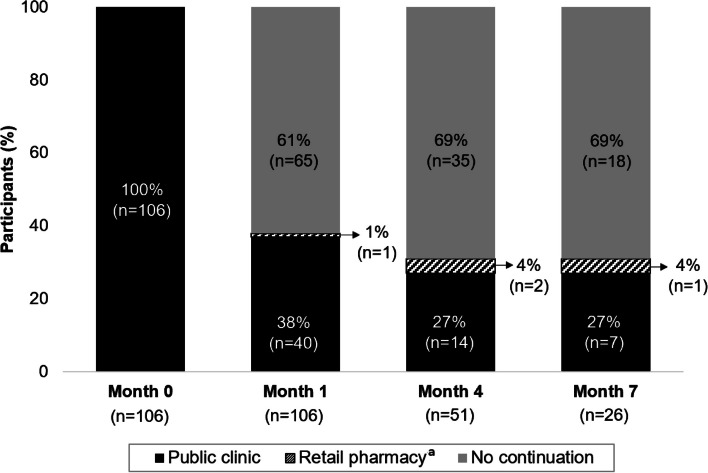
PrEP location selection and continuation at one, four, and seven months following initiation among pilot participants. Percentages are calculated amongst those eligible to initiate or continue PrEP at each visit. Month (M). ^a^ Participants who refilled at the pharmacy (*n* = 3 unique individuals) also refiled at a clinic at least once during the pilot. Only one such individual refilled twice at the pharmacy (at Months 4 and 7)

Over the duration of the pilot study (292 months of total participant follow-up), 39% (41/106) of participants refilled PrEP at any point. PrEP continuation was greatest at one month (38%, 40/106), then decreased by four (31%, 16/51) and seven (31%, 8/26) months, Fig. 1. Only three of the 41 participants (7%) that returned for any PrEP refills did so at a private pharmacy. Two of these participants only refilled PrEP once at a pharmacy (at one month and four months) and one refilled PrEP twice at a pharmacy (at four months and seven months); all these participants additionally refilled PrEP at a clinic either before (*n* = 2) or after (*n* = 2) they refilled PrEP at a pharmacy. Among the participants that continued PrEP, adherence was high. At one month, the median number of pills missed was 0 (IQR 1–2) and the ability to use and frequency of use scores were 100 (IQR 75–100); these findings remained consistent over PrEP follow-up visits.

In our subgroup analysis where we assessed any PrEP continuation by participants’ stated preference for PrEP access location at enrollment, we did not find any significant differences in continuation among those who reported a preference for accessing PrEP at clinics (43%, 23/53) versus pharmacies (40%, 18/48; *p* = 0.82).

At PrEP initiation and follow-up visits, most participants (> 50%) indicated that they were “unsure” of the extent to which they agreed or disagreed with the statements on the acceptability and appropriateness of pharmacy-delivered PrEP services, Table 2. At initiation, less than half of participants agreed or strongly agreed that they anticipated they would like (42%, 44/106) or recommend (43%, 46/106) pharmacy-delivered PrEP services; far below our a priori acceptability assessment threshold of > 80%. At follow-up visits, these findings remained consistent, with < 20% of participants agreeing or strongly agreeing with the acceptability assessment statements. Similarly, at one month (the first timepoint at which appropriateness was assessed), only 12% (5/41) of participants agreed or strongly agreed that pharmacy-delivered PrEP services fit their needs or was a good match for their needs; findings that remained consistent (and below our a priori appropriateness assessment threshold) at four and seven months.

**Table 2:** Implementation outcomes for the pharmacy-based PrEP refill intervention over the pilot duration

	**Initiation** **(*N* = 106)**	**Month 1** **(*N* = 41)**	**Month 4** **(*N* = 16)**	**Month 7** **(*N* = 12)**
**Acceptability**^**a**^				
“Like pharmacy-based PrEP delivery.”				
* Strongly agree or agree*	44 (42%)	5 (12%)	2 (13%)	2 (17%)
* Unsure*	62 (58%)	36 (88%)	14 (88%)	10 (83%)
“Would recommend pharmacy PrEP to others.”				
* Strongly agree or agree*	46 (43%)	5 (12%)	2 (13%)	2 (17%)
* Unsure*	60 (57%)	36 (88%)	14 (88%)	10 (83%)
**Appropriateness**^**b**^				
“Pharmacy-delivered PrEP fits my needs.”				
* Strongly agree or agree*	-	5 (12%)	2 (13%)	2 (17%)
* Unsure*		36 (88%)	14 (88%)	10 (83%)
“Pharmacy-delivered PrEP is a good match for my needs.”				
* Strongly agree or agree*	-	5 (12%)	2 (13%)	2 (17%)
* Unsure*		36 (88%)	14 (88%)	10 (83%)
**Costs**				
Any willingness to pay for pharmacy-based PrEP refills	88 (83%)	32 (78%)	12 (75%)	8 (67%)
Amount willing to pay				
(KES), median (IQR)	200 (100–300)	150 (100–300)	125 (100–250)	450 (250–550)
(USD^c^), median (IQR)	$1.81 (0.90–2.71)	$1.35 (0.90–2.71)	$1.81 (0.90–2.71)	$4.06 (2.26–4.97)

*Abbreviation: IQR* Interquartile range

^a^Our assessment of acceptability, a multi-faceted construct, was based on two components of the construct as defined by the Theoretical Framework of Acceptability (TFA): affective attitude (likes and recommendations to a friend) and burden (perceptions of ease and complication)

^b^Our assessment of appropriateness was based on the Intervention Appropriateness Measure (IAM) and Proctor’s definition of appropriateness. Appropriateness was only measured at follow-up visits

^c^Converted from Kenya Shillings (KES) to US dollars (USD) using conversion rate averaged from 11/2020 to 12/2021 ($1USD = 110.72 KSH)—https://www.exchangerates.org.uk/KES-USD-spot-exchange-rates-history-2020.html

Despite these findings, most participants (83%, 88/106 at initiation) reported that they would be willing to pay some amount for a package of pharmacy-delivered PrEP services, Table 2. At PrEP initiation, the median amount participants were willing to pay was 200 KES (IQR 100–300 KES), which is equivalent to ~ $2 USD (IQR ~ $1 to $3 USD). This reported amount decreased slightly at one and four months, then increased at seven months among participants still engaged in PrEP services.

From our qualitative data, we identified five primary reasons why interviewees did not opt to refill PrEP at a pharmacy (Table 3): convenience, cost, desire for continuity of the client-provider relationship, quality of care concerns, and misunderstanding the pharmacy PrEP refill option. First, some interviewees reported that getting PrEP at clinics was more convenient, as they could access other health services needed while there. Second, some interviewees said that the cost of pharmacy-delivered PrEP services deterred them from seeking PrEP refills at a pharmacy. Third, a few interviewees explained that they had established a relationship with PrEP providers at the clinic during their initiation visit and wished to continue seeing the same providers for follow-up, rather than new providers at the pharmacy. Fourth, a handful of interviewees expressed concern that pharmacy-based PrEP services would be lower quality in terms of provider competency and forthrightness, worrying, for example, that pharmacy providers would not maintain confidentiality. Lastly, despite having enrolled in the pilot study, two interviewees said they were unaware of the pharmacy PrEP refill option.

**Table 3:** Reasons why interviewees did not refill PrEP at pharmacies, with illustrative quotes

**Reason 1: Convenience of clinic-based PrEP refills**	
Clinic-based refills convenient when getting other health services at the same time	• *“When you go to the clinic, … you can get other services [you need at the same time], compared with going to the chemist.” (Female participant, age 24)* • *“[At the clinic,] every time that I have come for the PrEP, I have always been counselled together with [my partner at the that same time she gets her HIV treatment]. And probably every time I am tested yeah that is out of my good will.” (Male participant, age 28)*
**Reason 2: Cost of pharmacy-delivered PrEP services**	
Preference to get PrEP for free at clinic vs. paying for it at pharmacies	• *“In the chemist, I would have to pay for it [PrEP]. And [at] the hospital, it would be given for free. That, too, was a first priority [i.e., key consideration].” (Female participant, age 24)* • *“Just imagine that you are using your money to buy medication instead of food. You see it will be difficult [to pay for PrEP at the pharmacy].” (Female participant, age 41)*
**Reason 3: Desire for continuity of the client-provider relationship at clinic**	
Preference to continue seeing same PrEP providers started with at clinic	• *“You know, these [clinic-based providers] are people that I had already familiarized with. So, on coming to the hospital, they already know what I want.” (Male participant, age 32)* • *“[PrEP provider name redacted] has been refilling my PrEP. She is very friendly, and I have no issue with going to the clinic. So, I prefer [getting PrEP at the clinic] because she is very much friendly to me. We are happy with the services she is offering.” (Male participant, age 28)* • *“I went and met [clinic-based PrEP provider name]. She is a good woman. So, I thought, ‘What if I go to the chemist [to refill PrEP]? Will I find someone [at the pharmacy] who is as good as [clinic-based PrEP provider name]?” (Female participant, age 45)*
**Reason 4: Concerns about quality of pharmacy-delivered PrEP services**	
Mistrust of private pharmacy providers	• *“The problem with the chemist … [is] confidentiality. You know, most providers in the chemist, you may not trust them. Most of them are not doctors. Some will give you fake medicine, expired medication, or even medication that should not be sold to people … like that.” (Male participant, age 29)* • *“You know, the person [working] at the chemist, that is just a business. So he might be having greed for money, compared to these other doctors at the clinic.” (Male participant, age 31)*
Perceived lower quality of care at pharmacies	• *“[I prefer getting PrEP at a clinic] because in a chemist, anyone can go and work. … But for you to work in a hospital, you are supposed know…Let’s say you work under HIV-related issues; you must have the knowledge. You need to know what PrEP is, how it helps, its side effects, and how you can manage them. But in a chemist, an individual can be told, ‘Come and work for me.’” (Female participant, age 24)* • *“[I prefer getting PrEP at a clinic] because in the pharmacies, you may find that even the cleaning personnel is told to attend to a client.” (Male participant, age 29)* • *“You know, when you go to the clinic, they will listen to you. But when you go to the chemist, they are after your money.” (Female participant, age 24)*
Concerns about privacy and confidentiality at pharmacies	• *“About privacy, you know, most chemists do not have rooms like in hospitals. It is just like a kiosk. So when you go [to the pharmacy,] you say, ‘Give me.’ …It is even difficult to buy condoms [at the pharmacy].” (Male participant, age 29)* • *“At first, I felt insecure to … [get PrEP at] the chemist because, at that chemist, I have been their customer twice before. So, I was like, ‘If I could get [PrEP] there, and they know that [I’m taking PrEP], how would it be?” (Male participant, age 35)*
**Reason 5: Misunderstanding of intervention**	
Unaware of option to refill PrEP at study pharmacy	• *“I only knew about [PrEP’s availability at] government hospitals.” (Male participant, age 29)* • *“It wasn’t really a decision [to refill PrEP at a healthcare facility] because I just did not have much of that information [about the option to refill PrEP at a pharmacy]. So I just went [i.e. returned for refills] where I had begun [taking PrEP].” (Female participant, age 34)*

## Discussion

Findings from this mixed-methods implementation study suggest that individuals who initiate PrEP at public clinics may prefer accessing services there and that giving such clients the option to refill PrEP at nearby private pharmacies may do little to improve PrEP continuation among this demographic. Although PrEP continuation among participants in this pilot was comparable to that observed in other clinic-based PrEP implementation studies [38–40], almost no participants opted to refill PrEP at nearby private pharmacies. Most participant expressed uncertainty as to whether they thought pharmacy-delivered PrEP services would be acceptable and appropriate, which may have been driven by their lack of first-hand experience with this new PrEP delivery model. Participants’ concerns around the cost and quality of pharmacy-delivered PrEP refills may have further driven their low selection of this refill option, as could have a general lack of knowledge of this option among some participants.

Few participants may have selected pharmacy PrEP refills in this study because those enrolled may have already overcome barriers to clinic-delivered PrEP services and did not want to reinitiate PrEP care in a new service delivery location. We also did not implement any demand generation strategies to entice clients who might otherwise not initiate clinic-based PrEP services without a pharmacy-based refill option. As indicated by some interviewees, the clients we enrolled might have had a preexisting preference for clinic-delivered services because they or their sexual partners (especially those living with HIV) were already accessing other services, such as family planning or antiretroviral therapy, there. Due to the relatively short duration of the pilot (13 months), it is also possible that participants might not have selected to refill PrEP at pharmacies because this option was time-limited, which would have required them to reengage in clinic-delivered PrEP services eventually if they had long-term PrEP continuation goals.

The low uptake of pharmacy-delivered PrEP refills could also be partially attributable to the novelty and limited scale of this PrEP delivery model. Because pharmacy-delivered PrEP services are not widespread in Kenya and only available at a handful of pharmacies participating in research studies, participants had reason to be skeptical about the quality of pharmacy PrEP services (e.g., pharmacy provider competency) delivered in this new setting – especially considering there is currently no governmental guidelines, formal training curriculum, or oversight for pharmacy providers to deliver PrEP services. Additionally, our training of clinic-based PrEP providers on the option of pharmacy PrEP refills might not have been adequate, resulting in some providers not being aware of or fully understanding how this option worked, which could have resulted in incomplete information on the option being relayed to potential participants.

When the findings from this study are compared with those from two recently completed pilot studies in Kenya, which tested a model in which trained pharmacy providers both initiated and continued clients on PrEP with great success [18, 19], the evidence suggests that individuals who seek health services at public clinics may not be exchangeable with those who seek health services at private pharmacies. Modifications to our model where private pharmacies only dispense PrEP refills may be needed to potentially improve PrEP continuation outcomes, reach new PrEP clients, and decongest overburdened public clinics. Potential modifications could include adding demand generation strategies that target private pharmacies for the recruitment of new PrEP clients (who are then referred to public clinics for PrEP initiation), further subsidizing the cost of pharmacy PrEP refills (i.e., with client vouchers), or layering interventions that support linkage between clinics and pharmacies (e.g., client navigation services). These modifications may be needed because despite the demonstrated the feasibility of a stand-alone model of pharmacy-delivered PrEP initiation services [18, 19], in many settings, including Kenya, current policies (e.g., pharmacy provider scope of practice) do not allow task shifting PrEP prescribing and HIV testing to pharmacy providers.

This study had some limitations. First, we only implemented the pilot at two public clinics and three private pharmacies in Kenya, thus limiting the generalizability of our findings to other settings within and outside of Kenya. Second, our verbal referral to nearby pharmacies for PrEP refills may have been too simplistic; clinic-based PrEP clients interested in pharmacy refills may have benefited from a more formalized referral process supported with structured forms or financial incentives. Third, since most study participants did not refill PrEP at pharmacies, our observed implementation outcomes measured participants’ perceived acceptability and appropriateness of a model they never experienced; perceptions are often subject to change after individuals experience an intervention unfamiliar to them [41]. Fourth, all PrEP adherence outcomes were self-reported and thus subject to social desirability bias; they also were primarily reflective of clinic-based PrEP service delivery because few participants selected pharmacy-based refills. Fifth, our qualitative findings only reflected the perspectives of those who refilled PrEP at a clinic and not those who never returned for clinic-based PrEP refills and may have preferred a pharmacy-based refill option. Finally, since much of the pilot implementation occurred during the COVID-19 pandemic, our outcomes on selected location for PrEP refilling could have been biased by perceptions participants had about their safety and potential risk of COVID infection in these settings.

## Conclusions

The findings from this study emphasize the importance of developing client-centered models for PrEP service delivery that meet individuals who could potentially benefit from PrEP services where they are at and cater to their individual preferences. If individuals have a strong preference for the delivery of health services in a particular setting, instead of referring them elsewhere, we should continue to develop interventions that meet their needs and update supporting policies and supply chains to enable and facilitate these interventions. In Africa, private pharmacies are staffed by trained healthcare professionals and frequently deliver sexual and reproductive health services; pharmacy-based HIV prevention and treatment interventions could reach new populations who could benefit from these services and bring us closer to ending the AIDS epidemic [4].

### Supplementary Information

**Supplementary Material 1.**

**Supplementary Material 2.**

**Supplementary Material 3.**

## Data Availability

The datasets used and/or analyzed during the current study are available from the corresponding author on reasonable request.

## Abbreviations

AIDS: Acquired immunodeficiency syndrome
COVID-19: Coronavirus Disease 2019
HIV: Human immunodeficiency virus
IDI: In-depth interview
IQR: Interquartile range
KES: Kenyan Shilling
PrEP: Pre-exposure prophylaxis
RDT: Rapid diagnostic testing
SMS: Short message service
USD: United States dollar
