# Supplementary material for: Low selection of HIV PrEP refills at private pharmacies among clients who initiated PrEP at public clinics: findings from a mixed-methods study in Kenya
Source: BMC Health Serv Res. 2024 May 11;24:618. doi: 10.1186/s12913-024-10995-0 (PMC11088131; doi:10.1186/s12913-024-10995-0)
Supplement: Supplementary file 2 — Supplementary Material 2. [file 12913_2024_10995_MOESM2_ESM.docx]

# Additional file 2. Questionnaire: Follow-up

| This baseline questionnaire is to be conducted by a study counselor stationed at the pharmacy (Aim 1a) or HIV clinic (Aim 2). This baseline questionnaire has 15 parts: 1) demographics, 2) health seeking behaviors, 3) sexual behaviors, 4) fertility intentions, 5) medical history, 6) alcohol/substance abuse, 7) depression screening, 8) HIV risk perceptions, 9) general self-efficacy assessment, 10) HIV stigma, 11) PrEP perceptions, 12) pharmacy-based PrEP, 13) PrEP use, 14) COVID-19 questions, and 15) social harm reports. Participants should do their best to answer all questions and let the researcher know if they feel uncomfortable or prefer not to answer any of the following questions. | | |
| --- | --- | --- |
| **0. Logistics** | | |
|  | Visit date | [Captured in CommCare] |
|  | Participant ID: | *\|__\|__\|-\|__\|__\|-\|__\|__\|__\|-\|__\|-\|__\|* |
| **1. Demographics**  *First I would like to ask you some basic questions about yourself.* | | |
| 10. | Participants’ relationship status? | Single, no partners at all  Casual partner(s) only  One primary partner only  One primary partner and casual partners  Other…………….. |
| 11. | In the last three months, have you been verbally, physically or economically abused by a sexual partner? | *Yes [complete Social Harm Report]*  *No*  *Prefer not to answer* |
| **2. Health seeking behaviors** | | |
| 4. | [Aim 1a]: What brought you to the pharmacy for care today?  [Aim 2]: The last time you went to the pharmacy, what brought you into care? | *PrEP*  *Family planning*  *STI treatment*  *UTI treatment*  *Cold/cough*  *Sexual performance enhancing drugs*  *Treatment for chronic condition (e.g., diabetes, hypertension)*  *Seeking health for someone else*  *Other: ____________* |
| 5. | [Aim 1a]: How much did you spend at your pharmacy visit today?  [Aim 2]: Roughly how much did you spend at your last pharmacy visit? | *KSH: __________* |
| 6. | [Aim 1a]: How would you rate the quality of the services you received at the pharmacy today?  [Aim 2]: How would you rate the quality of services you received at your last pharmacy visit? | *Excellent*  *Good*  *Fair*  *Poor*  *Very poor* |
| 7. | Since the beginning of the COVID-19 outbreak, have you purchased anything at the pharmacy to prevent or treat COVID-19? | *Yes*  *No* |
| 7a. | [If Q7==Yes]: What have you purchased? [Check all that apply] | *Choloroquine*  *Azithromycin*  *Hand sanitizer*  *Other: ____________* |
| 9. | Since the beginning of the COVID-19 outbreak, have you changed how often you visit a public or private health clinic care? | *Yes, have increased visits*  *Yes, have decreased visits*  *No change in pharmacy visits* |
| 10. | [Aim 1a]:What brought you to the health clinic for care at your last visit?  [Aim 2]: What brought you to the health clinic for care today? | *PrEP*  *Family planning*  *STI treatment*  *UTI treatment*  *Cold/cough*  *Sexual performance enchancing drugs*  *Treatment for chronic condidition (e.g., diabetes, hypertension)*  *Seeking health for someone else*  *Other: ____________* |
| 11. | [Aim 1a]: How much did you spend at your clinic visit today?  [Aim 2]: Roughly how much did you spend at your last clinic visit? | *KSH: __________* |
| 12. | [Aim 1a]: How would you rate the quality of the services you received at your clinic visit today?  [Aim 2]: How would you rate the quality of the services you received at your last clinic visit? | *Excellent*  *Good*  *Fair*  *Poor*  *Very poor* |
| 13. | During the COVID-19 outbreak, have you had any trouble accessing general health care? | *Yes*  *No* |
| **3. Sexual Behaviors**  *Now I would like to ask you some questions about your sexual behaviors.* | | |
| 1. | In the past month, how many individuals have you had sex with? | *\|__\|__\| number of sexual partners*  *(if 0, go to Q3)*  *Prefer not to answer* |
| 1a. | Of these individuals, how many are new sexual partners? | *\|__\|__\| number of new sexual partners*  *Prefer not to answer* |
| 1b. | Of these individuals, how many do you think are living with HIV? | *\|__\|__\| number of sexual partners living with HIV*  *Prefer not to answer* |
| 1c. | How has COVID-19 impacted the number of individuals you had sex with in the past month? | *Increased number of partners*  *Decreased number of partners*  *No change* |
| 2. | In the past month, how many times did you have sexual intercourse? | *\|__\|__\| number of sex acts*  *(if 0, go to Q4)*  *Prefer not to answer* |
| 2a. | How many times was a condom used? | *\|__\|__\| number of times condom used*  *Prefer not to answer* |
| 2b. | How has COVID-19 impacted the number of times you have had sexual intercourse in the past month? | *Increased frequency of sex*  *Decreased frequency of sex*  *No change* |
| 3. | In the past 6 months, did you ever exchange sex for money or a gift? | *Yes*  *No*  *Prefer not to answer* |
| 4. | In the past 6 months, did you ever exchange money or a gift for sex? | *Yes*  *No*  *Prefer not to answer* |
| 5. | In the past 6 months, have you been diagnosed with an STI? | *Yes*  *No*  *Prefer not to answer* |
| 6. | In the past 6 months, have you used post-exposure prophylaxis (PEP)? | *Yes*  *No*  *Prefer not to answer* |
| 7. | In the past 6 months, have you had sex under the influence of drugs or alcohol? | *Yes*  *No*  *Prefer not to answer* |
| 8. | In the past 6 months, have you used injection drugs with shared needles and/or syringes? | *Yes*  *No*  *Prefer not to answer* |
| 9. | What (if anything) do you do to protect yourself from getting HIV? [Check all that apply] | *Use condoms*  *Abstain from sex*  *Limit number of sexual partners*  *Take PEP after sexual exposure*  *Take PrEP [prior to today]*  *Other: _______________* |
| 10. | In the past 2 weeks, how much has COVID-19 impacted your sexual activity? [Check all that apply] | *You have sex with your main partner more frequently*  *You have sex with your main partner less frequently*  *You have sex with a causal sex partner more frequently*  *You have sex with a casual sex partner less frequently*  *You exchange sex more frequently*  *You exchange sex less frequently*  *No change in sexual activity*  *Other:________________* |
| **4. Fertility intentions**  *Now I am going to ask you some questions about your fertility intentions* | | |
| 3. | Are you using any of the following birth control methods? [Mark all that apply] | *None*  *Oral*  *Implants*  *Post-Menopausal*  *IUD*  *Tubal Ligation/Hysterectomy*  *Pregnant*  *Injectable*  *Condoms*  *Other, specify: ___________* |
| 4. | Are you (and your partner) currently trying to conceive a child? | *Yes*  *No* |
| 5. | During the COVID-19 outbreak, have you or your partner had trouble accessing contraception? | *Yes*  *No* |
| **5. Medical history** | | |
| 1. | Have you ever tested for HIV? [prior to today] | *Yes*  *No* |
| 1a. | [If Q4==”Yes”] How many months ago was your last HIV test? [prior to today] | *Months: ________* |
| 1b. | [If Q4==”Yes”] How did you last test for HIV? | *Clinic-based: rapid test*  *Pharmacy-based: rapid test*  *Pharmacy-based: self-test (assisted)*  *Pharmacy-based: self-test (unassisted)*  *Other: _______________* |
| 1c. | How has your HIV testing been affected by COVID-19? | *Not at all*  *Challenges in accessing HIV testing, but tested*  *Has not tested* |
| 2. | Signs and symptoms of PrEP side effects? | *Yes*  *No* |
| 2a. | [If Q9==”Yes”] Mark all PrEP symptoms that apply: | *Headache*  *Rash*  *Nausea*  *Vomiting*  *Loss of appetite*  *Other:_______* |
| 2b. | [If Q9==”Yes”] Have any of these side effects been severe? | *Yes*  *No* |
| 2c. | [If Q9==”Yes”] Have any of these side effects affected your ability to consistently take PrEP? | *Yes*  *No* |
| **6. Alcohol use**  *Now I’m going to ask you about your alcohol use in the past year. While some of this information may be embarrassing or difficult to remember, please try to give your best answers and be as honest as possible.*  SOURCE: AUDIT-C | | |
| 1. | How often do you have a drink containing alcohol? | *Never*  *Monthly or less*  *2 to 4 times a month*  *2 to 3 times a week*  *4 or more times a week* |
| 2. | [If Q1!=”Never”] How many drinks containing alcohol do you have on a typical day when you are drinking? | *1 or 2*  *3 or 4*  *5 or 6*  *7 to 9*  *10 or more* |
| 3. | [If Q1!=”Never”] How often do you have six or more drinks on one occasion? | *Never*  *Less than monthly*  *Monthly*  *Weekly*  *Daily or almost daily* |
| 4. | [If Q1!=”Never”] In the past 1 month, did you have a drink containing alcohol just before or during sex? | *Yes*  *No* |
| 5. | In the past 1 month, has your partner been drunk from alcohol? | *Yes*  *No*  *N/A I don’t have a partner* |
| 6. | [If Q1!=”Never”] How has your alcohol use been affected by COVID-19? | *Increased alcohol intake*  *Decreased alcohol intake*  *No change* |
| **7. Depression screening (PHQ-9)**  *The next set of questions are a list of problems that people can get. These questions are about how you have been feeling* ***during the past two weeks.*** *For each item, please let me know if you have felt this or experienced this not at all; several days; more than half the days; or nearly every day.* | | |
| 1. | Little interest or pleasure in doing things | *Not at all*  *Several days*  *More than half the days*  *Nearly every day*  *Prefer not to answer* |
| 2. | Feeling down, depressed, or hopeless | *[See responses above]* |
| 3. | Trouble falling or staying asleep, or sleeping too much | *[See responses above]* |
| 4. | Feeling tired or having little energy | *[See responses above]* |
| 5. | Poor appetite or overeating | *[See responses above]* |
| 6. | Feeling bad about yourself – or that you are a failure or have let yourself or your family down | *[See responses above]* |
| 7. | Trouble concentrating on things, such as reading the newspaper or watching television | *[See responses above]* |
| 8. | Moving or speaking so slowly that other people could have noticed? Or the opposite – being so fidgety or restless that you have been moving around a lot more than usual | *[See responses above]* |
| 9. | Thoughts that you would be better off dead or hurting yourself in some way | *[See responses above]* |
| 10. | *[If participant checked any problems in Q1-9]* How **difficult** have these problems made it for you to do your work, take care of things at home, or get along with other people? | *Not difficult at all*  *Somewhat difficult*  *Very difficult*  *Extremely difficult*  *Prefer not to answer* |
| **8. HIV risk perceptions**  *Now I am going to ask you some questions about your beliefs surrounding risk of HIV infection. While some of this information might be difficult to answer or embarrassing, please try to give your best answer and be as honest as possible.* | | |
| 1. | Do you think PrEP makes sex completely safe from HIV? | *Yes*  *No*  *Maybe* |
| 2. | Are you generally a person who takes risks? I am going to read three answer choices and would like you to tell me which one is closest to the truth. | *I take risks*  *I avoid taking risks*  *I am somewhere in between* |
| 3. | In the past 3 months, how often have you thought about getting HIV? | *Never*  *Rarely*  *Some of the time*  *Often* |
| 4. | How worried are you about getting HIV in the next three months? | *No worry*  *Some worry*  *A lot of worry* |
| **9. General self-efficacy assessment**  *Now I am going to ask you a number of statements about yourself. After each statement, I want you to tell me if you think the statement is “not true at all”, “hardly true”, “moderately true” or “exactly true”.*  SOURCE: Schwarzer R, Jerusalem M. (1995) General self-efficacy scale. In Weinman J, Wright S, Johnston M, Measures in health psychology: A user’s portfolio. Causal and control beliefs (pp. 35-37). Windsor, UK: NFER-NELSON | | |
| 1. | I can always manage to solve difficult problems if I try hard enough. | *Not true at all*  *Hardly true*  *Moderately true*  *Exactly true* |
| 2. | If someone opposes me, I can find the means and ways to get what I want. | *[See responses above]* |
| 3. | It is easy for me to stick to my aims and accomplish my goals. | *[See responses above]* |
| 4. | I am confident that I could deal efficiently with unexpected events. | *[See responses above]* |
| 5. | Thanks to my resourcefulness, I know how ot handle unforeseen situations. | *[See responses above]* |
| 6. | I can solve most problems if I invest the necessary effort. | *[See responses above]* |
| 7. | I can remain calm when facing difficulties because I can rely on my coping abilities. | *[See responses above]* |
| 8. | When I am confronted with a problem, I can usually find several solutions. | *[See responses above]* |
| 9. | If I am in trouble, I can usually think of a solution. | *[See responses above]* |
| 10. | I can usually handle whatever comes my way. | *[See responses above]* |
| **10. HIV stigma**  *This section deals with feelings you might have if you were to acquire HIV, despite taking PrEP. We would like to know what you think regardless of whether these things would actually happen. Please tell me if you strongly agree; agree; disagree; or strongly disagree with the following four statements.*  If I acquired HIV, …. | | |
| 1. | I would be treated badly at work or get fired. | *Strongly agree*  *Agree*  *Disagree*  *Strongly disagree* |
| 2. | I would lose friends. | *[See responses above]* |
| 3. | My family would disown or neglect me. | *[See responses above]* |
| 4. | My community would treat me like an outcast. | *[See responses above]* |
| **11. PrEP perceptions** | | |
| 1. | Do you know of anyone else taking PrEP? | *Yes*  *No* |
| 1a. | *[If Q1==”Yes”]* Who do you know taking PrEP? *Check all that apply* | *Sexual partner*  *Friend*  *Family*  *Other: _____* |
| 2. | Does anyone know you are taking PrEP? | *Yes*  *No* |
| 2a. | [If Q2==”Yes”] Who knows you are taking PrEP? *Check all that apply* | *Sexual partner*  *Friend*  *Family*  *Other: _____* |
| 2b. | [If Q2==”Yes”] Overall, how supportive is these individual (these individuals) of you taking PrEP? | *Not very supportive*  *Moderately supportive*  *Very supportive* |
| 3. | Do you agree with the following statement: *“My future health depends on me taking PrEP”*? | *Strongly agree*  *Agree*  *Uncertain*  *Disagree*  *Strongly disagree* |
| 3a. | In the past month, have you encouraged a friend to start, find out about, or use PrEP? | *Yes*  *No* |
| *We understand that it is challenging to take a pill every day. Most people miss from time to time.* | | |
| 4. | Do you have concerns about being able to take PrEP every day? | *Yes*  *No* |
| 5. | How worried are you about getting side effects from PrEP? | *Not worried*  *Somewhat worried*  *Very worried* |
| ***PrEP Stigma***  *The following questions refer to general feelings people in your community may have about PrEP. Again, I am not referring to any specific person taking PrEP and would like to know what you think regardless of whether these things actually happen.*  *SOURCE: Modified perceived stigma (Kaai, Sahara-J: Journal of Social Aspects of HIV/AIDS, 2012).* | | |
| 6. | People in my community think people who take PrEP are promiscuous (having casual sex). | *Strongly agree*  *Agree*  *Disagree*  *Strongly disagree* |
| 7. | People in my community think people who take PrEP are being responsible about their sexual health. | *[See responses above]* |
| 8. | People in my community think PrEP may not be safe for your health. | *[See responses above]* |
| 9. | People in my community think that taking PrEP means you have HIV. | *[See responses above]* |
| 10. | I worry about people treating me differently because of my PrEP use. | *[See responses above]* |
| 11. | I feel bad about my PrEP use because of what people think of me. | *[See responses above]* |
| **12. Pharmacy-based PrEP**  *Now I would like to ask you some questions about your perceptions (Aim 2)/experiences (Aim 1a) with pharmacy-based PrEP delivery.* | | |
| 1. | [Aim 1a:] Overall, how would you rate your experience with pharmacy-based PrEP delivery? | *Excellent*  *Good*  *Fair*  *Poor*  *Very poor* |
| 2. | [Aim 1a:] Roughly how long did it take from the pharmacy provider initiating PrEP to you receiving your PrEP medication (including counseling & HIV testing)? | *Minutes: ___________* |
| 3. | Where you counseled on the importance of PrEP adherence and potential PrEP side effects? | *Yes*  *No* |
| 3a. | [If Q3=’Yes’:] Where did this counseling occur? | *Pharmacy: over the counter*  *Pharmacy: private back room*  *Pharmacy: public waiting room*  *Clinic: private room*  *Clinic: public space*  *Other: _____________* |
| 4. | Did you test for HIV today? | *Yes*  *No* |
| 4a. | [If Q4=’Yes’:] How did you test for HIV today? | *Clinic-based: rapid test*  *Pharmacy-based: rapid test*  *Pharmacy-based: self-test (assisted)*  *Pharmacy-based: self-test (unassisted)*  *Other: _______________* |
| 4b. | [If Q4=’Yes’:] Where did you test for HIV today? | *Pharmacy: over the counter*  *Pharmacy: private back room*  *Pharmacy: public waiting room*  *Clinic: private room*  *Clinic: public space*  *Other: _____________* |
| 4c. | [If Q4=’Yes’:] How comfortable did you feel testing for HIV in this setting? | *Very comfortable*  *Comfortable*  *Neutral*  *Uncomfortable*  *Very uncomfortable* |
| 4d. | [If Q4=’Yes’:] What were the results of your HIV test today? | *HIV-negative*  *HIV-positive*  *Indeterminant* |
| 5. | Are you likely to continue PrEP after your first month supply is finished? | *Very likely*  *Likely*  *Unlikely*  *Very unlikely* |
| 5a. | [If Q10=Very/likely:] Where do you plan to go to refill PrEP? | *Retail pharmacy*  *HIV clinic*  *Faminly planning clinic*  *Don’t know* |
| 6. | How much did you pay to initiate PrEP at this pharmacy (Aim 1a)/clinic (Aim 2) visit? | *KSH: ______________* |
| 7. | For this pilot, PrEP is being subsidized by the Kenya MOH but this might not be sustainable long term.  In the future, how much might you be willing to pay to access HIV testing + counseling + a 3-month supply of PrEP at a retail pharmacy? | *KSH: ______________* |
| **13. PrEP use**  *Now I would like to ask you some questions about your PrEP use.* | | |
|  | Have you decided to continue taking your PrEP medication? | *Yes*  *No* |
| 1a. | [If Q1=’No’] Why have you choose to discontinue PrEP? | *Partnership ended*  *Not having sex*  *Change in sexual behavior*  *Confidentiality/stigma*  *Too difficult to take PrEP daily*  *Thought tablets caused side effects*  *HIV infected*  *Other:_____________* |
| 1b. | [If Q1=’No’] How long ago (in days) did you discontinue taking PrEP? | *Days: ________* |
| 2. | In the past month, on how many days did you NOT take a PrEP tablet? | *Days: ________* |
| 3. | Please rate your ability, over the past month, to take the PrEP tablets exactly as you were instructed. | *Excellent*  *Good*  *Fair*  *Poor*  *Very poor* |
| 4. | In the past month, how often did you take your PrEP they way you were supposed to? | *Always*  *Usually*  *Sometimes*  *Rarely*  *Never* |
| 5. | In the past month, what caused you not to take your PrEP tablets? [Mark all that apply] | *N/A (missed no tablets)*  *Forgot*  *Illness*  *Did not have food*  *Alcohol use*  *Not having sex*  *Traveled*  *Change in routine*  *Lost tablets*  *Confidentiality/stigma*  *Prefer to take just before or after sex*  *Missed a visit and had no tablets*  *Shared tablets with someone else*  *Tablets stolen*  *Side effects*  *Other:_________________* |
| 6. | Since your last study visit, was there any period of time (>1 week) during which your sexual partner was away (e.g., traveling for work? | *Yes*  *No*  *Not applicable* |
| 6a. | [If Q4=’Yes’] Did you continue to take PrEP while your partner was away? | *Yes*  *No* |
| 7. | Everyone has stress in their lives; has taking PrEP affected the stress in your life? | *Less stress*  *No effect*  *More stress* |
| 8. | Has taking PrEP affected the pleasure in your sexual relationship(s)? | *Less pleasure*  *No effect*  *More pleasure* |
| 9. | Has your PrEP access to PrEP been affected by the COVID-19 outbreak? | *Yes*  *No* |
| 10. | Has your PrEP use been affected by the COVID-19 outbreak? | *Yes*  *No* |
| **14. COVID-19 Questions**  *Now I would like to ask you some questions about COVID-19* | | |
| 1. | How concerned do you feel about the novel coronavirus, COVID-19? | *Not concerned at all*  *A little concerned*  *Moderately concerned*  *Very concerned*  *Extremely concerned* |
| 2. | Have you made any changes to your lifestyle or daily activities because of COVID-19? | *Yes, I have made some changes to my lifestyle or daily activities*  *No, I have not changed my lifestyle or daily activities; I am doing everything I normally do* |
| 2a. | [If Q2=”Yes”] Which of the following? [Check all that apply] | *More hand washing and cleaning*  *Avoiding social gatherings*  *Avoiding gym/exercise classes*  *Avoiding going to the clinic for routine appointments*  *Avoiding going to the pharmacy*  *Avoiding going to the market*  *Working from home*  *Avoiding or canceling trips*  *Stocking up on food and supplies*  *Other: _________________* |
| 3 | Have you experienced any difficulties due to COVID-19? [Check all that apply] | *Reduced wages or work hours*  *I have lost my job*  *Childcare*  *Getting food*  *Getting hand sanitizer, soap, face mask or cleaning supplies*  *Getting routine/essential services*  *Transportation*  *Accessing healthcare*  *Other: ________________* |
| 4. | Please select your top 3 concerns today: [Select 3] | *Not being able to put food on the table*  *Not being able to get medical care*  *Not being able to work*  *Not being able to take care of family members*  *I have no concerns*  *Other: ______________* |
| *In an effort to reduce the spread of COVID-19, many are practicing social distancing and self-isolation. Self-isolation is the act of staying away from situations where you may be in close contact with others, such as social gatherings, work, school, faith-based gatherings, sports gatherings, restaurants and other public gatherings.* | | |
| 5. | To what extent are you social isolating? | *All of the time. I stay home nearly all of the time.*  *Most of the time. I only leave my house to buy good and other essentials*  *Some of the time. I have reduced the amount of times I am in public spaces, social gatherings, or at work*  *None of the time. I am doing everything I normally do.*  *I am limiting my social interactions to family members who live in my community.* |
| *In an effort to reduce the spread of COVID-19, many are wearing face masks to cover their nose and mouth whenever they are out of their houses.* | | |
| 6. | How often are you wearing a face mask? | *All of the time.*  *Most of the time.*  *Some of the time.*  *None of the time.* |
| 7. | Are you currently taking any medication(s) to prevent COVID-19 infection? | *Yes*  *No* |
| 7a. | [If Q7==”Yes”] What medication(s) are you taking? | *____________________* |
| 8. | What sources do you trust to provide accurate COVID-19 information? [Select all that apply] | *Official government announcements on the radio/TV*  *Facebook*  *Newspapers*  *Friends or family members*  *Healthcare professionals*  *Twitter*  *County health department*  *Other: ________________* |
| 9. | In the past 2 weeks, how concerned have you been about COVID-19 in your neighborhood? | *Not concerned at all*  *Somewhat concerned*  *Very concerned* |
| *Mental health screening during COVID-19* | | |
| 10. | In the past 7 days, how often have you felt nervous, anxious or on edge? | *Not at all or <1 day*  *1-2 days*  *3-4 days*  *5-7 days* |
| 11. | In the past 7 days, how often have you felt depressed? | *[See responses above]* |
| 12. | In the past 7 days, how often have you felt lonely? | *[See responses above]* |
| 13. | In the past 7 days, how often have you felt hopeful about the future? | *[See responses above]* |
| 14. | If there are medications and/or vaccines developed with potential to treat and/or prevent COVID-19 would you be willing to participate in a study to ascertain their efficacy and/or safety? | *Yes*  *No*  *I don’t know* |
| **15. Social Harm Report**  *[This portion of the questionnaire will only appear for individuals who reported any social harm above. The following questions are not interviewer-administered. Please collect the following information in a culturally appropriate way in the context of a counseling session.]* | | |
| 1. | In the last 3 months, was the participant **verbally** abused by an intimate/sexual partner? | *Yes*  *No*  *Prefer not to answer* |
| 1a. | [If Q1=’Yes’] How often was the participant **verbally** abused by his/her intimate partner (last 3 months)? | *\|__\|__\| number of times*  *Prefer not to answer* |
| 1b. | [If Q1=’Yes’] Was the **verbal** abuse related to study participation? | *Yes*  *No* |
| 2. | In the last 3 months, was the participant **physically** abused by his or her intimate partner? | *Yes*  *No*  *Prefer not to answer* |
| 2a. | [If Q2=’Yes’] How often was the participant **physically** abused by his or her intimate partner (last 3 months)? | *\|__\|__\| number of times*  *Prefer not to answer* |
| 2b. | [If Q2=’Yes’] Was the **physical** abuse related to study participation? | *Yes*  *No* |
| 3. | In the last 3 months, was the participant **economically** abused by his or her intimate partner? | *Yes*  *No*  *Prefer not to answer* |
| 3a. | [If Q3=’Yes’] How often was the participant **economically** abused by his or her intimate partner (last 3 months)? | *\|__\|__\| number of times*  *Prefer not to answer* |
| 3b. | [If Q3=’Yes’] Was the **economic** abuse related to study participation? | *Yes*  *No* |
| 4. | Was the participant abused by his or her intimate partner in any other way (not fitting into the categories of verbal, physical, or economic abuse)? | *Yes (If Yes, describe in Q6)*  *No*  *Prefer not to answer* |
| 4a. | Concisely describe the social harm: | *Prefer not to answer*  *Explain: ______________* |
| 5. | Was the abuse from the participant’s primary/main sexual partner? | *Yes*  *No* |
| 5a. | [If Q5==’No’] Describe the nature of the relationship to the intimate partner: |  |
| 6. | What were the consequences of the abuse? [Mark all that apply] | *None*  *Relationship break-up*  *Loss of income or economic support*  *Loss of employment*  *Change of residence*  *Loss of custody of children*  *Prefer not to answer*  *Other: ______________* |
