# Supplementary material for: Low selection of HIV PrEP refills at private pharmacies among clients who initiated PrEP at public clinics: findings from a mixed-methods study in Kenya
Source: BMC Health Serv Res. 2024 May 11;24:618. doi: 10.1186/s12913-024-10995-0 (PMC11088131; doi:10.1186/s12913-024-10995-0)
Supplement: Supplementary file 3 — Supplementary Material 3. [file 12913_2024_10995_MOESM3_ESM.docx]

# Additional file 3. In-Depth Interview Guide: Clients

**Interviewer instructions: Administer informed consent. Once signed, begin guide.**

| **0.0 Interview Information**  ***Fill out items A through F prior to starting interview.*** | |
| --- | --- |
| 1. Informed consent has been administered: YES / NO   *If consent form has not been signed by participant,*  *interview must not proceed.*   1. Interview ID: _____________________________   *Format: Interviewee Type-DDMMYY-Number interview conducted that day*  *where “U” = PrEP user and “P” = peer of PrEP users (i.e., PrEP non-user)*  *(e.g., “U-021220-02” represents the second PrEP user interviewed on 12 Feb 2020)* | |
| 1. Date of interview: ____/_____/_______   *Format: DD/MM/YYYY* |  |
| 1. Location of interview: __________________________________________ | |
| 1. Interviewer’s full name: _________________________________________ | |
| 1. Interview start time: ____________________   *Format: HH:MM am or pm* | |
| 1. Interview end time: _____________________   *Format: HH:MM am or pm* | |

**Facilitator introduction: [DO NOT READ; GUIDE ONLY]**

Hello. My name is _______, and I am a ________, working at _______. Thank you for taking the time to talk with me today.

The purpose of this interview is to understand your experiences with pharmacy-based PrEP delivery and future interest in pharmacy-based PrEP delivery.

There are no right or wrong answers to these questions. People have different views and we are interested to learn more about these experiences from you. Today, you are in the role of a teacher and I am here to learn from you since you are an expert in your own life experiences and opinions.

This interview should take around an hour to complete. Please let me know if at any time you have questions, if something I say is not clear, or if you need to take a break.

| 1. **[*INTERVIEWER: Mark the participant’s sex.*]** | *Male*  *Female* |
| --- | --- |
| ***START INTERVIEW HERE.*** | |
| I’d like to start off by asking you some basic questions about yourself. | |
| 1. How old are you? | _________ years old |
| 1. What is the highest **level** of school you have attended? | *Pre-primary*  *Primary*  *Post-primary, vocational*  *Secondary/‘A’ level*  *College/post secondary vocational school*  *University*  *Don’t know* |
| 1. How many **years** did you complete at that level? | ______ years |
| 1. What is your current occupation? | _________________________ |
| 1. About how much money do you make per month? | _______ KSH per month |
| 1. What is your current marital status? | *Married*  *In a relationship, not married*  *Single, never married*  *Single, divorced or widowed* |
| 1. Thinking about everyone you live with, about how much money per month does your household make as a whole? | _______ KSH per month |
| 1. Do you have any living children? | *Yes*  *No* |
| 1. **[If Q9=YES]** How many living children do you have? | *__________* children |

| **Theme** | **Sub-Theme** | **Question** |
| --- | --- | --- |
| **Services received** | **All clients: Basic PrEP Journey Details** | I’d like to start off by asking you some basic questions about your PrEP use.  For how long have you been using PrEP?  Did you initiate PrEP at the pharmacy or at the CCC? *(If “CCC”, skip over the next section of questions)* |
|  | **Initiators: How Heard about PBP**  ***Anticipated Effectiveness* (*Relative Advantage)* of PBP Initiation** | ***[Ask the questions in this section only of initiators, meaning participants who initiated PrEP at a study pharmacy.]***  How did you hear that you could start PrEP at this pharmacy?  Why did you decide to initiate PrEP at a pharmacy rather than elsewhere, like at a Comprehensive Care Center?  *Probes:*   - Was there anything in particular that made you want to get PrEP at a pharmacy rather than another location? |
|  | **Initiators: Experience of PrEP Counseling & HIV Risk Assessment**  ***(Intervention Coherence, Ethicality, Knowledge & Beliefs)*** | ***[Again, ask the questions in this section only of initiators, meaning participants who initiated PrEP at a study pharmacy.]***  I’d like you to think only about the pharmacy visit during which you started PrEP. So for now, think only of that first visit and not any other times you came for PrEP refills. Ok? *[Pause for participant to indicate that s/he has understood the instructions.]*  Great. Could you please describe for me what happened during that first visit when you started PrEP?  ***IMPORTANT****: Probe as needed to get details on the remaining questions in this section. If participant starts to describe HIV testing, feel free to ask the HIV testing-related questions-- from the next section at this point.*  Did the pharmacy provider give you information about PrEP?   - If so, what information did he or she tell you? - Did the information the pharmacy provider gave you make sense to you? (Did you find anything confusing about the information the pharmacy provider gave you?) - Where in the pharmacy did the two of you talk? *(Probe as needed to understand if it was over the counter where other customers might be able to overhear the conversation, in a totally private and enclosed room, in a semi-private space that was not completely enclosed, such as behind some shelves, etc.)* - Thinking about the place where the two of you talked, did you feel as though it had enough privacy? Why or why not?   Did the pharmacy provider ask you about your sexual activity, such as whether you recently had engaged in condomless sex?  If so, did you feel comfortable discussing your sexual activity with the pharmacy provider? Why or why not? *(If participant reports discomfort, probe to understand if discomfort was due to the space they were in, characteristics of the pharmacy provider such as sex or age, or any other particular aspect of how PrEP was delivered to them.)*  Have you ever refilled PrEP at a pharmacy? ***(If no, skip to the section “HIV Test Experience”)*** |
|  | **Refill-only clients:**  **How Heard about PBP** | ***[For refill-only clients, meaning clients who initiated PrEP elsewhere but got PrEP refills at a study pharmacy.]***  How did you hear that you could get your PrEP refilled at this pharmacy? |
|  | **All clients who’ve ever refilled:**  ***Anticipated Effectiveness* (*Relative Advantage)***  **of PBP Refills** | How many times have you refilled your PrEP at the pharmacy?  Why did you decide to refill PrEP at a pharmacy rather than elsewhere, like at a Comprehensive Care Center?  *Probes:*   - Was there anything in particular that made you want to get PrEP at a pharmacy rather than another location? |
|  | **All clients:**  **HIV Test Experience** | During your pharmacy visits to get PrEP, did you ever do an HIV test?  *If no, confirm:*  So the pharmacy provider never required you to do an HIV test in order to get PrEP? If so, why not?  *If yes:*  Did you do an HIV self-test at the pharmacy? *(If needed, explain: “The self-test is one where you collect your own blood or saliva sample, put it in a solution, and interpret the results either on your own or with the assistance of a trained professional.)*  *If “no” to self-testing:*  Can you describe the HIV test you received?  *Probes:*  Did the pharmacy provider take a sample of blood? How long did it take for you to learn the results?  *If “yes” to self-testing:*  Before taking the HIV self-test, did the pharmacy provider explain to you what the test entailed, including what would happen in the event of a positive result?  *If yes:*  What did you think about the pharmacy provider’s explanation of the HIV self-test?  *Probes:*   - Did their explanation of the HIV self-test make sense to you? (Did you find any of it confusing?)   Overall, how was your experience doing the HIV self-test?  *Probes:*   - What did or didn’t you like about it? - How easy or hard was it for you to do the self-test? Please explain.   Did the pharmacy provider help you interpret the results?  *If yes:*   - How did you feel about the way the pharmacy provider explained the HIV test results to you?   *Probes:*   - Were you able to understand the pharmacy provider’s explanation? If no, what was difficult to understand? |
|  | **All clients who’ve ever refilled:**  **Adherence & Side Effects *(Burden)*** | ***[Ask all clients the following questions, regardless of whether they initiated PrEP at the pharmacy or elsewhere]***  Since starting PrEP, were there ever days when you missed or forgot to take your PrEP pill?  *If no, confirm:*  So you’ve always managed to take your PrEP pill, without missing any days?  *If yes:*  How many times would you say you missed or forgot to take your PrEP pill?  Was there anything that caused you to miss or forget to take your PrEP pill? *(Probe to understand what barriers to adherence they faced, such as whether they had difficulties getting back to the pharmacy for a refill, or difficulties getting into the habit of taking a daily pill, etc.)*  Since starting PrEP, have you ever experienced any side effects from PrEP, like nausea or headache?  *If yes:*  What side effects did you experience? For how long? |
|  | **All clients who’ve ever refilled:**  **Experience of Adherence Counseling & Side Effect Assessment** | Could you describe for me what happened during the pharmacy visits when you refilled PrEP?  ***IMPORTANT****: Probe as needed to get details on the questions below. If participant starts to describe HIV testing, feel free to ask the HIV testing-related questions from the next section at this point.*  Did the pharmacy provider ask you about your adherence to PrEP, meaning whether you had been taking it every day?  *If yes:*  Where in the pharmacy did this discussion take place? (*Probe as needed to understand if it was over the counter where other customers might be able to overhear the conversation, in a totally private and enclosed room, in a semi-private space that was not completely enclosed, such as behind some shelves, etc.)*  Did you feel as though this space had enough privacy? Why or why not?  In general, did you feel comfortable discussing your adherence with the pharmacy provider? Why or why not? *(If participant reports discomfort, probe to understand if discomfort was due to the space they were in, characteristics of the pharmacy provider such as sex or age, or any other particular aspect of how PrEP was delivered to them.)*  *(If participant said earlier that s/he missed PrEP on some days)* Did the pharmacy provider give you any advice on how to take PrEP every day without missing? If so, what? And did you find this advice useful? Why or why not?  Did the pharmacy provider ask whether you had experienced any side effects from taking PrEP, such as nausea or headache?  *If yes:*  In general, did you feel comfortable discussing side effects you had experienced with the pharmacy provider? Why or why not?  *(If participant reports having experienced side effects from PrEP)*  Where in the pharmacy did your discussion about side effects take place? *Probe as needed to understand if it was over the counter where other customers might be able to overhear the conversation, in a totally private and enclosed room, in a semi-private space that was not completely enclosed, such as behind some shelves, etc.)*  Did you feel as though this space had enough privacy? Why or why not?  Did the pharmacy provider do anything to help you deal with your side effects? If so, what? And did this help?  *Probes:*   - Did the pharmacy provider recommend any over-the-counter medicines to treat your side effects? - Did the pharmacy provider refer you to a health facility? If so, how did that referral go? |
|  | ***All clients:***  ***Burden,***  ***Opportunity Costs, & Relative Advantage*** | I’d now like to ask you some general questions about how you get PrEP.  About how long does it take you to get from your house to the pharmacy? *(Probe as needed to get an estimate in minutes/hours. Ensure that the time estimate is one way, not round-trip.)*  Does it cost you any money to travel from your house to that health care facility?  If yes:  About how much money does it cost you to go from your house to that health care facility one way? *(Probe as needed to get an estimate in KSH.)*  Overall, how easy or hard is it for you to get to the pharmacy to get your PrEP?  ***[For initiators only]*** About how much did the pharmacy charge you to start PrEP? *(Probe as needed to get an estimate in KSH.)*  ***[For clients who’ve ever refilled]*** About how much did the pharmacy charge you for a one-month refill PrEP? *(Probe as necessary to get an estimate in KSH.)* And about how much did the pharmacy charge you for a three-month refill PrEP? *(Probe as necessary to get an estimate in KSH.)*  In general, how do you feel about the price this pharmacy charges for PrEP?  *Probes:*   - Do you think they are too high, too low, or just right? Please explain. *(Probe as needed to understand if there are particular parts of the price—such as the consultation fee—that they think should be different. If they think the price is too high or too low, probe to understand why they think this/what they base this judgement on.)* |
| **General Pros and Cons of PBP** | *Relative Advantage* | ***(Only ask if not already answered*)**  Overall, do you think offering PrEP at private pharmacies **benefits** clients in any way? If so, please explain.  *Probes:*   - Why might a client choose to get PrEP at a private pharmacy rather than elsewhere?   Do you think offering PrEP at private pharmacies has any **negative consequences** for clients? If so, please explain.  *Probes*   - Why might a client choose to get PrEP elsewhere—like at a public hospital—rather than at a private pharmacy? |
| **Future Interest in PBP** | Desired Changes  &  Future Interest | Overall, how satisfied are you with the PrEP services you receive at this pharmacy?  *Probes:*   - If one of your friends wanted to start PrEP, would you recommend that they get it from a private pharmacy? Why or why not?   Is there anything about how the pharmacy delivers PrEP that you think could be improved? If so, what? *(Probe as needed to understand what, exactly, they’d like to see changed, what difference they think this change would make, and for whom/who would benefit from this change.)*  Do you plan to continue taking PrEP? Why or why not? *(If “no”, probe to understand their reason for stopping PrEP, e.g., change in risk, don’t like side effects, etc.)*  *If yes:*  Will you continue getting PrEP from a private pharmacy? Why or why not? |
| **COVID-19 impact** |  | How concerned do you feel about the novel coronavirus, COVID-19?  How has your lifestyle changed as a result of COVID-19?  *Probes:*   - How have your daily activities changed?   Describe any hardships or difficulties you have faced during COVID-19.  How do you feel the COVID-19 outbreak affect your healthcare seeking behaviors in general? Including preventive health seeking behaviors, such as access to contraception.  Have you requested any medications for COVID-19 treatment or prevention at your pharmacy or clinic? If yes, which ones?  How do you feel the COVID-19 outbreak affected your ability to get PrEP at the pharmacy?  What are some of the advantages or disadvantages you see with the delivery of PrEP at pharmacies during the COVID-10 outbreak? |
| **Closing Thoughts** | - | Is there anything else you would like to share with me today about your experience getting PrEP from private pharmacies? |

We have come to the conclusion of the topics I had prepared to discuss today.

**THANK YOU FOR YOUR TIME!**

**[Mark interview end time on page 1 (Item F).]**
